# *Acinetobacter baumannii* complex-caused bloodstream infection in ICU during a 12-year period: Predicting fulminant sepsis by interpretable machine learning

**DOI:** 10.3389/fmicb.2022.1037735

**Published:** 2022-11-01

**Authors:** Jun Xu, Xiaojun Chen, Xia Zheng

**Affiliations:** ^1^Intensive Care Unit, The First Affiliated Hospital, College of Medicine, Zhejiang University, Hangzhou, China; ^2^Real Doctor AI Research Centre, Zhejiang University, Hangzhou, China

**Keywords:** *Acinetobacter baumannii* complex-caused bloodstream infection, fulminant sepsis, machine learning model, Shapley additive explanation, treatment strategies

## Abstract

**Background:**

*Acinetobacter* baumannii complex-caused bloodstream infection (ABCBSI) is a potentially fatal infection in intensive care units (ICUs). This study proposed an interpretable machine learning (ML) model to predict ABCBSI fulminant fatality.

**Methods:**

A retrospective study of ICU patients with ABCBSI was performed in China from 2009 to 2020. Patients were stratified into two groups: those that suffered from fulminant sepsis and died within 48 h, and those that survived for more than 48 h. The clinical score systems and ML models with Shapley additive explanation (SHAP) were used to develop the prediction models. The ML model was internally validated with five-fold cross-validation, and its performance was assessed using seven typical evaluation indices. The top 20 features ranked by the SHAP scores were also calculated.

**Results:**

Among 188 ICU patients with ABCBSI, 53 were assigned to the non-survival group and 135 to the survival group. The XGBoost model exhibited the greatest area under the receiver operating characteristic curve (AUC), which outperformed other models (logistic regression, AUC = 0.914; support vector machine, AUC = 0.895; random forest, AUC = 0.972; and naive Bayesian, AUC = 0.908) and clinical scores (Acute Physiology and Chronic Health Evaluation II (APACHE II), AUC = 0.855; Sequential Organ Failure Assessment (SOFA), AUC = 0.837). It also had a sensitivity of 0.868, a specificity of 0.970, an accuracy of 0.941, a positive predictive value of 0.920, a negative predictive value of 0.949, and an F1 score of 0.893. As well as identifying the top 12 different important predictors that contribute to early mortality, it also assessed their quantitative contribution and noteworthy thresholds.

**Conclusion:**

Based on the XGBoost model, early mortality in ABCBSI is estimated to be more reliable than other models and clinical scores. The 12 most important features with corresponding thresholds were identified and more importantly, the SHAP method can be used to interpret this predictive model and support individual patient treatment strategies.

## Introduction

Bloodstream infection (BSI) is a major cause of infectious disease morbidity and mortality, and typically refers to a patient with systemic signs and symptoms of infection who has a positive blood culture (Timsit et al., 2020). Patients in the intensive care unit (ICU) are particularly predisposed to BSI, with a prevalence of ~15.2% (Vincent et al., 2020). *Acinetobacter* baumannii complex (ABC) has a high potential for nosocomial transmission, particularly in the ICU. In 2017, carbapenem-resistant *Acinetobacter baumannii* was listed among the antibiotic-resistant “critical priority pathogens” by the World Health Organization (Tacconelli et al., 2018). ABC-caused BSI (ABCBSI) is a critical problem in the ICU as it can cause sepsis or septic shock, and prolonged hospital stays, thus increased costs and mortality rates (Guo et al., 2016; Russo et al., 2019). The 2021 Surviving Sepsis Campaign (SSC) guidelines suggested that early identification and appropriate management in the initial hours after the development of sepsis can improve outcomes (Evans et al., 2021). However, it is still unclear whether fulminant sepsis is more likely to result in higher mortality because of host- or treatment-related factors.

Prediction is common in the medical field, such as anticoagulation by risk scores, risk stratification of ICU patients, early-warning systems for sepsis, and superhuman imaging diagnostics (Chen and Asch, 2017). It is also common for clinicians to use regression analysis when testing causal hypotheses and recently, machine learning (ML) approaches have emerged from analyzing big data in medicine. Through learning the patterns of the health trajectories of large numbers of patients, the ML model can predict clinical events at an expert level, drawing from information well beyond the individual physician’s practice experience (Rajkomar et al., 2019). ML has been applied in several fields of ICU, with studies using big data to predict mortality in ICU patients, readmission, and the length of ICU stay, as well as the risks of developing sepsis and acute respiratory distress syndrome (ARDS; Gutierrez, 2020). Although ML models can provide more accurate predictions, they are still difficult to translate into medical practice, especially when applied to individual patients. One reason is that the ML model makes it harder to succinctly present or explain the subtle patterns behind a particular prediction, which is often called the “black box.” Thus, to better interpret changes in risk parameters on a continuous basis, we need an interpretable ML model to rationalize the quantitative relationship between clinical parameters and outcome predictions.

The rapid diagnosis and treatment of BSI patients are crucial to their prognosis since timely and effective infection treatment can significantly improve outcomes (Civitarese et al., 2017; Timsit et al., 2020). To early identify the potential risk factors which could predispose to a fulminant course of ABCBSI is essential, and it may help to provide an appropriate treatment to potentially reduce the risk of exacerbations. This study aimed to construct ML models to predict early mortality in ABCBSI and interpret the model using the Shapley additive explanation (SHAP) method so that the predictive model can not only predict the results but also provide reasonable explanations.

## Materials and methods

### Study population

This retrospective study was conducted in the First Affiliated Hospital, College of Medicine, Zhejiang University, from January 2009 to December 2020. All ICU adult patients (age ≥18 years) diagnosed with ABCBSI were considered. The exclusion criteria were: (1) positive blood cultures before ICU admission; (2) patients who were not the first infected and no patient was included twice; (3) positive blood cultures containing other pathogenic microorganisms. The study was approved by the hospital Ethics Committees (IIT20210605A) and there was no need for informed consent because of the retrospective nature of the study.

### Data collection and preprocessing of data

The following data were extracted from the patients’ medical records: demographic information, vital signs [temperature, mean arterial pressure (MAP), and PaO2/FiO2 (P/F) ratio], laboratory tests [white blood cells (WBCs), hemoglobin, platelets, albumin, alanine aminotransferase (ALT), aspartate aminotransferase (AST), bilirubin, creatinine, blood urea nitrogen (BUN), C-reactive protein (CRP), prothrombin time (PT), activated partial thromboplastin time (APTT), PH, bicarbonate, lactate, sodium, potassium, chloride] at the onset of ABCBSI, invasive procedures before the acquisition of BSI, antibiotic exposure, antimicrobial susceptibility, antimicrobial therapy (time of initiation, doses, routes), the Pitt bacteremia score, Acute Physiology and Chronic Health Evaluation II (APACHE II) and Sequential Organ Failure Assessment (SOFA), and outcome.

Some variables were measured more than once so their maximum, minimum, and average values were further analyzed as independent variables. The overall missing data rate was <0.05% among all the variables and average values were input for missing variables.

### Machine learning

The predictive model was based on ML algorithms with the input of variables that different (*p* < 0.1) in the univariate analysis between the non-survival and survival groups. Five ML algorithms were used: extreme gradient boosting (XGBoost), logistic regression (LR), support vector machine (SVM), random forest (RF), and naive Bayesian (NB). All analyses were performed using Python (version 3.9.10). The parameters of XGBoost can be divided into three types: general, booster, and task. General parameters define which kind of booster is used in the lifting process and the commonly used boosters are the tree model and linear model. This article uses a tree model, which is the default option. The maximum number of threads was defined as 6. The parameters for Tree Booster include the learning rate (eta = 0.01), the maximum depth of each tree (max_depth = 3), and the proportion of subsamples used to train the model in the whole sample set (subsample = 1). The main task was to solve a binary logistic regression problem (objective = binary: logistic). After building the model, the area under the receiver operating characteristics curve (AUC), sensitivity, specificity, accuracy, positive predictive value (PPV), negative predictive value (NPV), and F1 score were used as evaluation indicators of model performance. To select the optimal feature subset for the predictive model, 5-fold cross-validation was used for the training and validation set. Four of the five folds were used as the training set, and the remaining one was used as the validation set.

SHAP is a game-theoretic approach to explain the output of the ML model. It connects optimal credit allocation with local explanations using the classical Shapley values from game theory and their related extensions. Shapley values are a widely used approach from cooperative game theory with desirable properties. SHAP values are a unified approach for explaining the outcome of our ML model and provide consistent and locally accurate attribution values for each feature (Lundberg et al., 2018; Tseng et al., 2020).

### Statistical analysis

Continuous variables are expressed as mean ± standard, and categorical variables are expressed as proportions. The variables were compared by Student’s *t*-test, the Mann–Whitney test for continuous variables, and the *χ*^2^ test or Fisher’s exact test for categorical variables, respectively. A two-sided value of *p* < 0.05 was considered statistically significant. Python (version 3.9.10) was used for the statistical analysis and visualizations.

## Results

### Demographic and clinical characteristics

This study included 188 ICU patients with ABCBSI from 2009 to 2020 and their demographic and clinical characteristics are presented in Table 1. Overall, 28.2% (53/188) of patients with fulminant sepsis died within 48 h.

**Table 1:** Baseline characteristics.

Features	Survival (*n* = 135)	Nonsurvival (*n* = 53)	Value of *p*
Clinical parameters			
Age (year)	61.4 ± 17.6	59.5 ± 15.7	0.509
Male *n* (%)	99 (73.3%)	37 (69.8%)	0.627
Vital signs			
Temperature (°C)	38.7 ± 1.1	38.8 ± 1.0	0.294
MAP (mm Hg)	70.4 ± 12.2	59.4 ± 13.0	<0.001
P/F ratio	272.0 ± 131.2	130.5 ± 96.4	<0.001
Underlying diseases			
Hypertension	52 (38.5%)	19 (35.8%)	0.734
Diabetes mellitus	23 (17.0%)	9 (17.0%)	0.993
Solid-organ malignancy	26 (19.3%)	9 (17.0%)	0.718
CAD	21 (15.6%)	10 (18.9%)	0.582
CRF	22 (16.3%)	9 (17.0%)	0.909
Liver cirrhosis	8 (5.9%)	6 (11.3%)	0.338
COPD	25 (18.5%)	10 (18.9%)	0.956
Hematological malignancy	4 (3.0%)	9 (17.0%)	0.002
Cerebrovascular disease	14 (10.4%)	3 (5.7%)	0.465
CTD	14 (10.4%)	4 (7.5%)	0.554
Laboratory parameters			
WBCs (×10^9^ L^−1^)	14.5 ± 10.5	11.2 ± 12.0	0.070
Hemoglobin (g dL^−1^)	8.1 ± 2.0	8.5 ± 2.2	0.295
Platelets (×10^9^ L^−1^)	154.1 ± 132.1	58.0 ± 70.8	<0.001
Albumin (g L^−1^)	31.2 ± 4.7	29.0 ± 5.8	0.010
ALT (U L^−1^)	72.9 ± 125.9	121.8 ± 254.8	0.187
AST(U L^−1^)	76.8 ± 152.6	169.0 ± 438.7	0.140
Bilirubin (μmol L^−1^)	54.2 ± 97.0	71.2 ± 73.8	0.249
Creatinine (μmol L^−1^)	91.1 ± 86.3	123.7 ± 92.7	0.023
BUN (mmol L^−1^)	10.8 ± 6.4	16.5 ± 11.5	<0.001
CRP (mg L^−1^)	116.0 ± 74.2	166.0 ± 117.0	0.005
PT (s)	14.3 ± 3.2	18.3 ± 8.0	0.001
APTT (s)	47.3 ± 22.0	59.5 ± 29.7	0.008
pH	7.4 ± 0.1	7.2 ± 0.2	<0.001
Bicarbonate (mmol L^−1^)	24.0 ± 6.5	25.4 ± 34.6	0.762
Lactate (mmol L^−1^)	2.9 ± 2.1	6.9 ± 4.7	<0.001
Sodium (mmol L^−1^)	137.3 ± 6.5	141.7 ± 8.5	0.001
Potassium (mmol L^−1^)	3.8 ± 0.6	3.8 ± 0.7	0.977
Chloride (mmol L^−1^)	103.0 ± 6.2	106.3 ± 7.9	0.002
Invasive procedures			
Mechanical ventilation	110 (81.5%)	50 (94.3%)	0.026
Central venous catheter	102 (75.6%)	42 (79.2%)	0.591
CRRT	48 (35.6%)	23 (43.4%)	0.318
PICCO	14 (10.4%)	10 (18.9%)	0.116
Previous antibiotic used (within 1 month)			
Corticosteroid use	27 (20.0%)	16 (30.2%)	0.135
Anti-pseudomonal penicillins + beta lactamase inhibitors	81 (60.0%)	33 (62.3%)	0.775
Antipseudomonal cephalosporins	27 (20.0%)	16 (30.2%)	0.135
Aminoglycosides	8 (5.9%)	3 (5.7%)	1.000
Carbapenems	85 (63.0%)	42 (79.2%)	0.032
Quinolone	36 (26.7%)	21 (39.6%)	0.082
Tigecycline	15 (11.1%)	6 (11.3%)	0.967
Anti-fungal agents	50 (37.0%)	29 (54.7%)	0.027
Carbapenem-resistant strains	119 (88.1%)	52 (98.1%)	0.063
Concurrent infection with another pathogen	55 (40.7%)	18 (34.0%)	0.391
Septic shock	51 (37.8%)	42 (79.2%)	<0.001
Immunosupression	43 (31.9%)	31 (58.5%)	0.001
Appropriate empirical therapy	34 (25.2%)	7 (13.2%)	0.074
Length of ICU stay before BSI	15.0 ± 43.0	7.7 ± 10.1	0.224
Severity of illness			
CCI	2.3 ± 2.2	2.5 ± 2.4	0.555
APACHE II score^a^	22.1 ± 8.8	34.9 ± 9.3	<0.001
SOFA score^a^	8.8 ± 4.4	15.7 ± 5.0	<0.001
Pitt bacteremia score^a^	4.5 ± 2.9	7.5 ± 2.5	<0.001

Data are *n* (%) or mean ± SD. MAP, mean arterial pressure; P/F, PaO_2_/FiO_2_; CAD, coronary artery disease; CRF, chronic renal failure; COPD, chronic obstructive pulmonary disorder; CTD, connective tissue disorder; WBCs, white blood cells; ALT, alanine aminotransferase; AST, aspartate aminotransferase; BUN, blood urea nitrogen; CRP, C reactive protein; CRRT, continuous renal replacement therapy; PICCO, pulse index continuous cardiac output; ICU, intensive care unit; BSI, bloodstream infection; CCI, charlson comorbidity index; APACHE, acute physiology and chronic health evaluation; SOFA, sequential organ failure assessment. *p* < 0.05, which are considered statistically significant.

a At the onset of ABCBSI.

Compared to the survival group, the non-survival group was more likely to have hematological malignancy, prior exposure to carbapenems and anti-fungal agents, receive mechanical ventilation, have septic shock, immunosuppression, and higher clinical scores assessed by the Pitt bacteremia, APACHE II, and SOFA scores at the time of BSI. In addition, decreases in MAP, P/F ratio, platelets, and PH and elevated creatinine, BUN, CRP, PT, APTT, lactate, sodium, and chloride were associated with early death.

### Model building and evaluation

**Figure 1:**
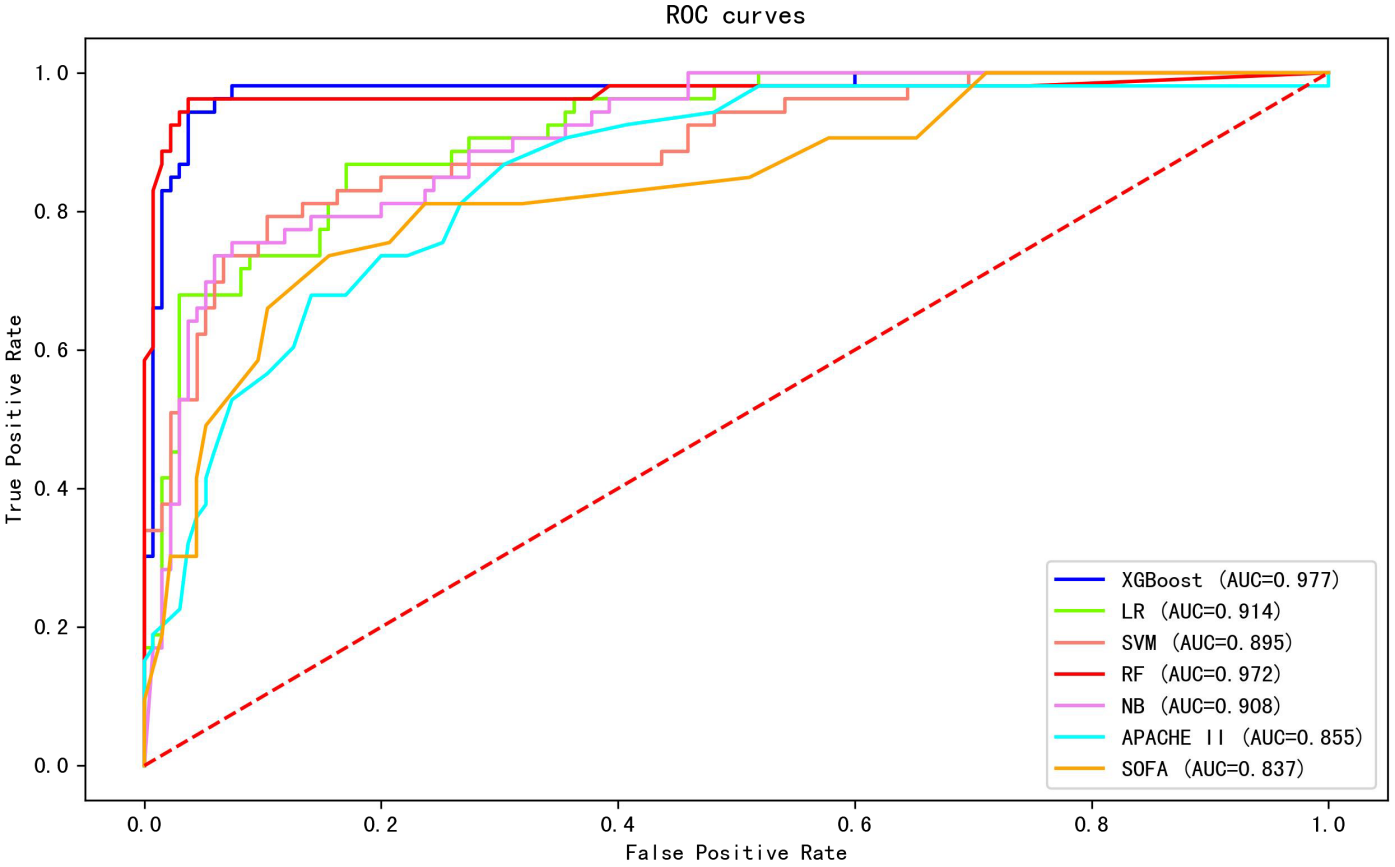
The AUC of different machine learning models and clinical scores in predicting early mortality. The results show that the XGBoost exhibited the largest AUC (0.977).

Twenty-six features (*p* < 0.1) in the univariate analysis between the two groups were chosen as the input variables in our ML model to predict early death. The results showed that the largest AUC (0.977) to predict early mortality was constructed by XGBoost. The XGBoost model performance was superior to other models (LR, AUC = 0.914; SVM, AUC = 0.895; RF, AUC = 0.972; and NB, AUC = 0.908) and the conventional clinical scores (APACHE II, AUC = 0.855; SOFA, AUC = 0.837; Figure 1).

The XGBoost model exhibited good performance by other evaluation indices, which included sensitivity of 0.868, a specificity of 0.970, an accuracy of 0.941, a positive predictive value of 0.920, a negative predictive value of 0.949, and an F1 score of 0.893. Table 2 shows the comparison of the predictive performance of different ML models.

**Table 2:** Comparison of the predictive performance of different ML.

Model	AUC	SE	SP	AC	F1 score	PPV	NPV
XGBoost	0.977	0.868	0.970	0.941	0.893	0.920	0.949
LR	0.914	0.375	0.900	0.789	0.429	0.500	0.844
SVM	0.895	0.375	0.933	0.816	0.462	0.600	0.848
RF	0.972	0.500	0.900	0.816	0.533	0.571	0.871
NB	0.908	0.625	0.933	0.789	0.556	0.500	0.893

ML, machine learning; AUC, The area under the receiver operating characteristic curve; SE, sensitivity; SP, specificity; AC, accuracy; PPV, positive predictive value; NPV, negative predictive value; XGBoost, extreme gradient boosting; LR, logistic regression; SVM, support vector machine; RF, random forest; NB, naive Bayesian.

### Explanation of risk factors

**Figure 2:**
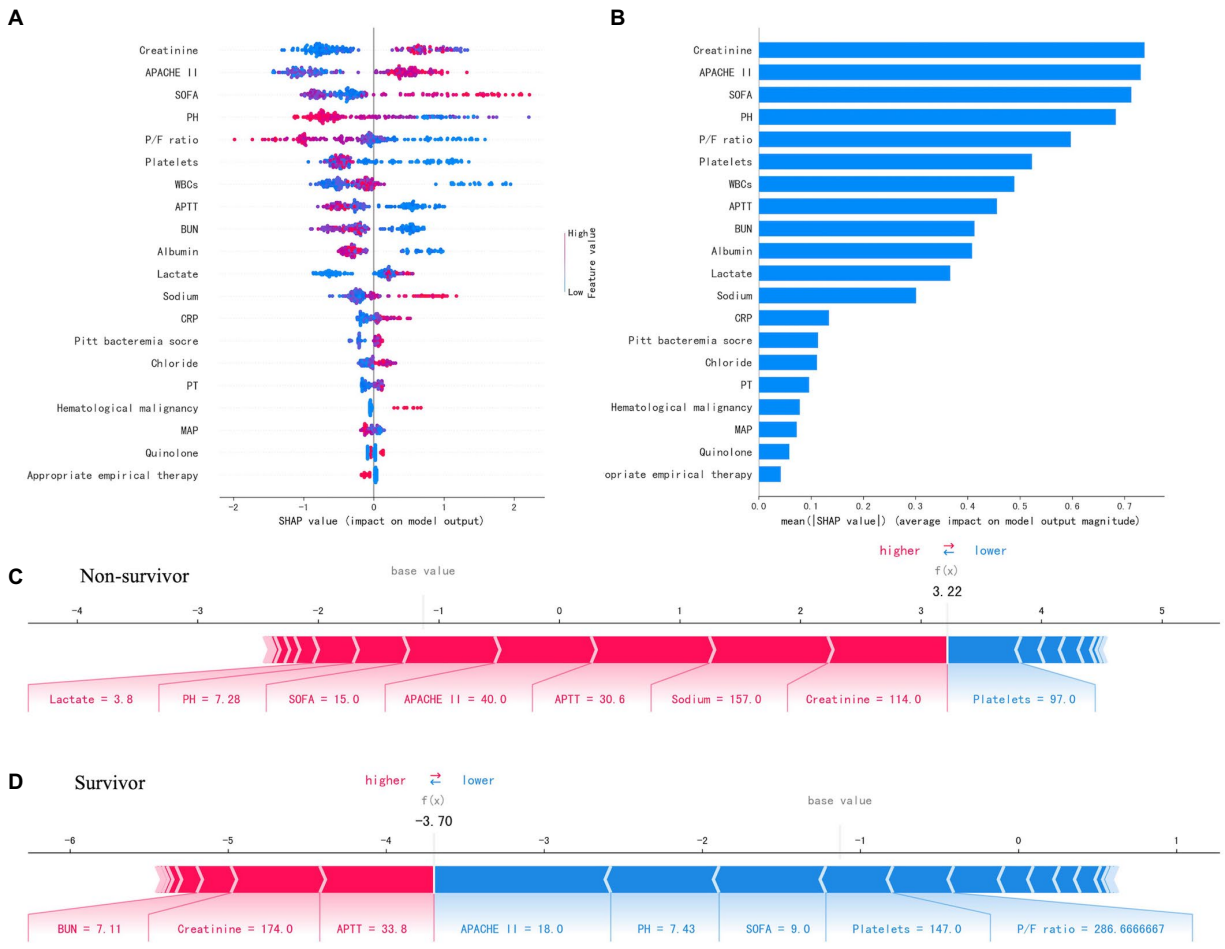
Feature analysis of the XGboost model. **(A)** A summary plot of the SHAP values for the top 20 features of our model. **(B)** The importance ranking of the top 20 variables according to the average absolute SHAP value. **(C,D)** The interpretation of model prediction results with the two samples. APACHE II, Acute Physiology and Chronic Health Evaluation II; SOFA, Sequential Organ Failure Assessment; P/F, PaO_2_/FiO_2_; WBCs, white blood cells; APTT, activated partial thromboplastin time; BUN, blood urea nitrogen; CRP, C-reactive protein; PT, prothrombin time; MAP, mean arterial pressure.

The SHAP summary was plotted for an overview of which features are most important for our XGBoost model. Figure 2A shows the top 20 risk factors in our model and the red color represents high feature value, while the blue color is the opposite. From top to bottom, the overall influence of features on the final prediction gradually decreases. For example, increases in creatinine have a positive impact and push the prediction toward mortality, whereas increases in PH have a negative impact and push the prediction toward survival. Figure 2B shows the top 20 important features evaluated by the average absolute SHAP value, the top 12 of which seem to be particularly important in our model. The level of creatinine had the strongest predictive value for all prediction horizons, followed closely by the APACHE II score, SOFA score, PH, and P/F ratio. Figures 2C,D show the individual force plots for patients who did not survive and survived, respectively. The red features (on the left) indicate increased mortality risk, and the blue features indicate decreased mortality risk. For example, this patient (Figure 2C) is predicted to have a 322% risk of a poor outcome due to the elevated creatinine (114 μmol L^−1^), sodium (157 mmol L^−1^), APTT (30.6 s), APACHE II score (40 points), SOFA score (15 points), and lactate (3.8 mmol L^−1^) level, and decreased PH (7.28). Creatinine is the most important risk-increasing variable and platelets (97 × 109 L^−1^) are the most important protective variable. The patient (Figure 2D) was predicted to survive due to a lower APACHE II score (18 points), SOFA score (nine points), and normal PH (7.43), platelets (147 × 109 L^−1^), and P/F ratio (286.7) level. The APACHE II score is the most important risk-decreasing variable.

**Figure 3:**
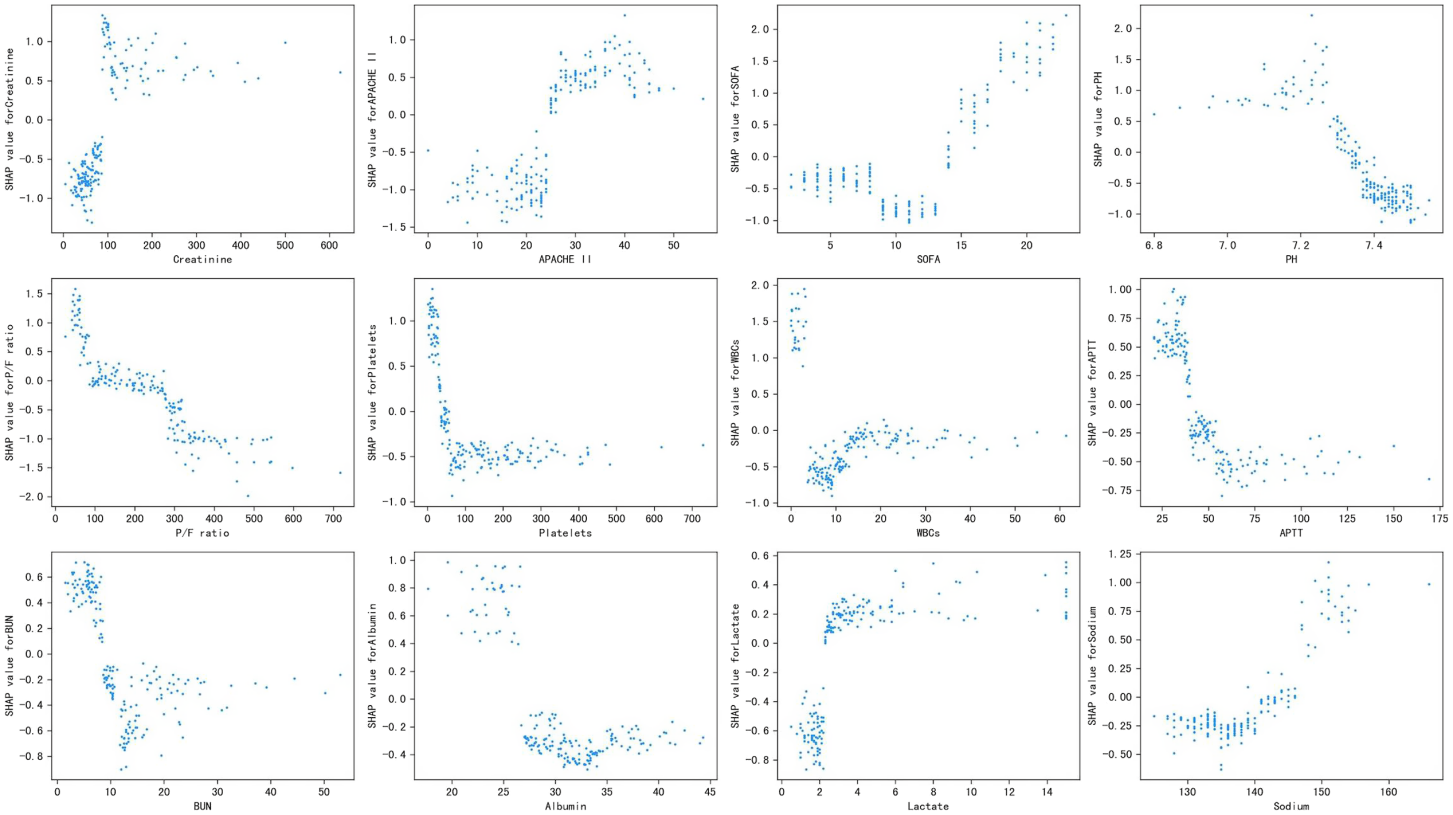
Partial SHAP dependence plot of the XGboost model. It shows how a single feature (the top 12 important variables) affects the output of the XGBoost predictive model. SHAP values for specific features exceed zero, representing an increased risk of death. APACHE II, Acute Physiology and Chronic Health Evaluation II; SOFA, Sequential Organ Failure Assessment; P/F, PaO_2_/FiO_2_; WBCs, white blood cells; APTT, activated partial thromboplastin time; BUN, blood urea nitrogen.

Figure 3 shows the SHAP dependence plot of the top 12 most important variables, showing that higher creatinine, APACHE II, SOFA, lactate, and sodium and lower PH, P/F ratio, platelets, WBCs, APTT, BUN, and albumin levels were related to higher mortality. The SHAP values for these features exceed zero, representing an increased risk of early mortality, so each feature has a cut-off point when a horizontal line is drawn.

## Discussion

*Acinetobacter baumannii* complex is a group of nosocomial pathogens and one of the six leading multidrug-resistant pathogens causing deaths in hospitals worldwide (Murray et al., 2022). It is responsible for a variety of clinical manifestations, of which ventilator-associated pneumonia (VAP) and BSI are the most common. ICU clinicians pay the most attention to BSI caused by ABC because it can cause sepsis and septic shock which are associated with more poor outcomes. A previous systematic review and meta-analysis including 10 studies reported that the pooled mortality of patients with ABCBSI was ~56.3% (Du et al., 2019). The mortality risks for ABCBSI include old age, malignancy, chronic renal disease, chronic liver disease, neutropenia, septic shock, immunosuppressant use, total parenteral nutrition, ICU stay, previous antibiotic use, Pitt bacteremia score, APACHE II score, SOFA score, lower albumin levels, bacteremia origin, carbapenem resistance, and inappropriate initial antimicrobial therapy (Du et al., 2019; Russo et al., 2019; Zhou et al., 2019; Son et al., 2020; Gu et al., 2021; Yu et al., 2021). However, previous studies mainly focused on testing hypotheses involving causal relationships and the predictive effect of conventional regression analysis methods may be unsatisfactory because it is mainly used to solve linear problems and is difficult to fit the real distribution of data. Therefore, it is important to obtain a more accurate predictive model for mortality and the decision-making process of the model must be understood by the physician. A recent study developed an ML model to predict patient outcomes of BSI based on electronic medical records and the model AUC was 0.81 using only 25 features (Zoabi et al., 2021).

In this study, we proposed an ML model using selected features for the prediction of ABCBSI fulminant fatality. The XGBoost model performed relatively better than other models (LR, SVM, RF, and NB) as well as conventional clinical scores (APACHE II and SOFA). Among the 26 selected features in our model, the top 12 important features with absolute SHAP values were creatinine, APACHE II, SOFA, PH, P/F ratio, platelets, WBCs, APTT, BUN, albumin, lactate, and sodium, which increased or decreased the risk of early mortality of ABCBSI to varying degrees. Furthermore, the SHAP summary plot of XGBoost revealed additional important features (e.g., creatinine, APACHE II score, platelets, WBCs, APTT, albumin, lactate, etc.) that logistic regression did not include. The well-established risk factors for mortality of ABCBSI, such as creatinine, albumin, APACHE II score, and lactate have been used as prognostic markers in several studies (Du et al., 2019; Russo et al., 2019) but other factors, such as APTT and platelets, are less used as predictors of outcome in ABCBSI. Therefore, the SHAP values were used to further illustrate whether each feature contributed positively or negatively to the target outcome.

The SOFA and APACHE II scores are the most commonly used methods and authoritative critical illness evaluation systems in ICU. According to a retrospective cohort study of ICU patients with suspected infections, defining sepsis by an increase in SOFA score provided more accurate prognoses (AUC, 0.753) than either SIRS criteria (AUC, 0.589) or qSOFA (AUC, 0.607; Raith et al., 2017). The APACHE II score classifies diseases based on the severity from 0 to 71, with higher scores representing more severe illnesses and greater mortality risks. The AUC of SOFA or APACHE II score is not high and is no more than 0.85, even though they have been proven useful prognostic biomarkers for critical illnesses (Tian et al., 2022). Many studies were analyzed using multivariable logistic regression methods, with the AUC ranging from 0.76 to 0.84 (Tseng et al., 2020). Recent studies have shown that ML models tend to have better predictive power than standard scoring systems (Morgan et al., 2019; Zhang et al., 2020). In line with these findings, our study demonstrated that the performance of the ML model was superior to the APACHE II and SOFA scores, in contrast to a systematic review that showed that logistic regression for the clinical prediction model is not inferior to the ML model (Christodoulou et al., 2019).

Our study not only generated a more accurate predictive model and identified other unrecognized key risk factors but also made it “explainable.” Each component of the predictive model can be visualized and contributes differently to the final outcome. Our study benefits from using SHAP values to uncover the black box of the ML model, therefore, our predictive model can provide implications for patient management, even when applied to individual patients. Additionally, based on the SHAP dependence plot, we further demonstrated the quantitative relationship of this contribution (Figure 3). Among the 12 most important features, most had a critical threshold at which the predicted risk abruptly changed. For example, the platelets <50 × 109 L^−1^ or lactate >2.5 mmol L^−1^ resulted in a significant increase in mortality risk. There were some unexpected situations, such as higher creatinine led to a higher risk of death, while higher BUN was protective. Although both serum creatinine and BUN can represent renal function, they are not completely consistent. Acute kidney injury (AKI) is defined by increased serum creatinine or decreased urine volume, which are significantly associated with mortality in sepsis (Peerapornratana et al., 2019), whereas the increase in BUN is not only affected by renal function, but also by stress-nutrition status and bleeding-volume status. A study reported a U-shape relationship between the BUN/creatinine ratio and all-cause mortality in the general population (Shen et al., 2022). Therefore, some important features may be missed due to the nonlinear relationship between features and risks in logistic regression. ML is particularly useful for handling enormous numbers of predictors, sometimes remarkably, more predictors than observations, and combining them in nonlinear and highly interactive ways (Obermeyer and Emanuel, 2016). Thus, the study offers a “warning threshold” that despite the parameters being in the normal reference range, the risk still increases in this model, and thus caution is needed.

This study has several limitations. First, it is a single-center retrospective study, so information bias and temporal bias should not be neglected. Second, the model was constructed with only a small number of patients, therefore, it needs to be externally validated in a multicenter study with a large sample size to determine its applicability. Third, in patients with other bacterial pathogens concomitantly isolated with ABC, it was not possible to judge whether the infection was caused by ABC or the concomitant pathogen(s), or both. Finally, this model was used on all patients admitted to the ICU but it needs to be tested on general wards as well and more external validation is required in the future.

## Conclusion

In conclusion, an interpretable ML model with optimal performance was constructed to predict early mortality in ABCBSI. Twelve of the most important features with corresponding thresholds crucial for early mortality prediction were identified. Furthermore, clinicans should be aware of important features (such as creatinine, APACHE II score, SOFA score, PH, P/F ratio, etc) beyond their corresponding thresholds, even within the normal range. However, this study needs to be confirmed in clinical settings and externally.

## Data availability statement

The data analyzed in this study is subject to the following licenses/restrictions: The datasets generated during and/or analyzed during the current study are available from the corresponding author on reasonable request. According to the national legislation and institutional requirements, patient data used in this study is confidential. To protect patient confidentiality and participant’s privacy, data used for this study can be obtained in an anonymous form only according to the data privacy act. Requests to access these datasets should be directed to XZ, zxicu@zju.edu.cn.

## Author contributions

JX contributed to data collection and manuscript writing. XC contributed to the data analysis. XZ contributed to the study design and revise the manuscript. All authors contributed to the article and approved the submitted version.

## Conflict of interest

The authors declare that the research was conducted in the absence of any commercial or financial relationships that could be construed as a potential conflict of interest.

## Publisher’s note

All claims expressed in this article are solely those of the authors and do not necessarily represent those of their affiliated organizations, or those of the publisher, the editors and the reviewers. Any product that may be evaluated in this article, or claim that may be made by its manufacturer, is not guaranteed or endorsed by the publisher.
